# Early Norepinephrine Attenuates Fluid-Associated Albumin Decline in Sepsis: A Prospective Longitudinal Study

**DOI:** 10.3390/jcm15093203

**Published:** 2026-04-22

**Authors:** Gianni Turcato, Arian Zaboli, Alessandra Eugenia Bionda, Michael Maggi, Fabrizio Lucente, Alberto Caregnato, Daniela Milazzo, Paolo Ferretto, Christian J. Wiedermann

**Affiliations:** 1 Intermediate Care Unit, Department of Internal Medicine, Hospital Alto Vicentino (AULSS7), 36014 Santorso, Italy; 2 Department of Health Sciences, UniCamillus–Saint Camillus International University of Health Sciences, 00131 Rome, Italy; 3 Health Professions Management, South Tyrolean Health Authority (SABES-ASDAA), 39100 Bolzano, Italy; 4 Department of Clinical and Experimental Medicine, University of Pisa, 56126 Pisa, Italy; 5 Institute of General Practice and Public Health, Claudiana College of Health Professions, 39100 Bolzano, Italy

**Keywords:** sepsis, serum albumin, cumulative fluid exposure, early norepinephrine, albumin trajectory, capillary leak, endothelial dysfunction, fluid stewardship, intermediate care unit

## Abstract

**Background/Objectives:** Hypoalbuminaemia is a consistent predictor of mortality in sepsis; however, the temporal dynamics of albumin decline and its relationship with fluid exposure and early norepinephrine therapy remain incompletely characterised. Determining whether early norepinephrine use is associated with attenuation of albumin loss could inform fluid management strategies and identify therapeutic windows for combined vasopressor–albumin interventions. The study aimed to assess whether serum albumin trajectories in sepsis are associated with fluid exposure, modulated by early norepinephrine therapy, and related to 30-day mortality. **Methods:** We conducted a prospective longitudinal study of patients admitted to an intermediate care unit (IMCU) with community-acquired sepsis. Serum albumin concentrations, cumulative fluid balance (CFB), and vasopressor use were recorded during the first 5 days of hospitalisation. Longitudinal mixed-effects and segmented linear models assessed the association of CFB and vasopressor therapy with albumin trajectories. Lagged mediation modelling explored the potential mediating role of albumin in the association between fluid exposure and 30-day mortality. **Results:** A total of 389 patients with community-acquired sepsis were included. Thirty-day mortality was 18%. Mean serum albumin at baseline was 2.58 g/dL and declined early to 2.24 g/dL at 72 h. Serum albumin was inversely correlated with cumulative fluid balance over time (r ranging from −0.235 to −0.348; *p* < 0.001). In longitudinal models, each 1% increase in ΔCFB was associated with a −0.029 g/dL decrease in serum albumin (*p* < 0.001), supporting an independent effect of fluid exposure. Before norepinephrine initiation, the albumin slope was −0.043 g/dL per interval and was −0.008 g/dL after vasopressor initiation (interaction *p* = 0.012). Lower albumin concentrations at 72 h predicted 30-day mortality (OR 1.49 per 0.5 g/dL decrease), and serum albumin mediated 18.6% of the association between fluid exposure and mortality. **Conclusions:** Cumulative fluid exposure was associated with a progressive decline in serum albumin in patients with community-acquired sepsis. Early norepinephrine initiation was associated with attenuation of this trajectory, consistent with the hypothesis that vasopressor-guided haemodynamic stabilisation may limit fluid-associated albumin loss.

## 1. Introduction

Sepsis remains a leading cause of mortality and is characterised by a dysregulated host response resulting in potentially life-threatening alterations in tissue perfusion [1,2]. Fluid resuscitation is a cornerstone of early management; however, its use remains controversial, as excessive fluid administration appears to be associated with worse outcomes [3,4].

Hypoalbuminaemia is common in patients with sepsis, and serum albumin is among the most consistent predictors of adverse outcomes [5]. During the acute phase of sepsis, serum albumin concentrations decline rapidly, reflecting a complex interplay of reduced hepatic synthesis, fluid-induced haemodilution, increased transcapillary escape, and interstitial redistribution [5,6,7]. In addition, serum albumin levels correlate with capillary permeability, and both the magnitude and temporal pattern of albumin decline may reflect the severity of endothelial injury, the adequacy of fluid resuscitation, and the evolution of tissue perfusion abnormalities [8,9].

More recently, earlier initiation of norepinephrine has emerged as a strategy to reduce fluid administration and minimise fluid-related harm [10,11]. Early restoration of vascular tone may also help contain increases in capillary permeability, thereby limiting albumin extravasation and pathological capillary leak [12]. Despite the established prognostic value of serum albumin and growing evidence supporting early norepinephrine use, the relative contributions of haemodilution and pathological capillary leak to albumin decline have not been disentangled in longitudinal studies. It also remains unclear whether norepinephrine modifies albumin trajectories through restoration of vascular tone, restriction of fluid exposure, or both. In addition, the association between serum albumin and mortality has been evaluated mainly using single-admission measurements, whereas its longitudinal trajectory, particularly in relation to fluid therapy and norepinephrine treatment, has not been adequately investigated [9,10,13].

If haemodynamic stabilisation achieved with norepinephrine modulates vascular permeability and attenuates albumin decline, this could provide a mechanistic rationale for early combined vasopressor–albumin strategies aimed at preserving intravascular oncotic pressure and limiting crystalloid exposure. Conversely, if albumin decline mediates the association between fluid exposure and adverse outcomes, albumin trajectories could serve as a dynamic marker to guide fluid de-escalation and identify patients who may benefit from targeted albumin supplementation [14,15,16].

### Objectives

The objectives of this study were to

(1) quantify the independent association between cumulative fluid exposure and serum albumin trajectories over the first 5 days of sepsis;
(2) assess whether early norepinephrine therapy modifies this association over time;
(3) determine whether albumin concentrations at clinically relevant timepoints independently predict 30-day mortality and whether albumin decline mediates the association between fluid exposure and adverse outcomes.

## 2. Materials and Methods

### 2.1. Study Design

We conducted a prospective longitudinal cohort study with serial assessment of serum albumin, cumulative fluid balance, and norepinephrine exposure at six prespecified time points over 5 days.

### 2.2. Study Setting and Patients

This study was conducted in the Intermediate Medical Care Unit (IMCU) of Alto Vicentino Hospital, Santorso, Italy, between 1 September 2023 and 31 March 2025. All consecutive adult patients admitted to the IMCU from the emergency department (ED) with a diagnosis of sepsis were screened for eligibility. Sepsis was defined according to Sepsis-3 criteria as suspected or documented infection associated with an increase of at least 2 points in the Sequential Organ Failure Assessment (SOFA) score [1].

Exclusion criteria were age younger than 18 years, known or suspected pregnancy, transfer from hospital wards other than the ED, postoperative or post-traumatic sepsis within the previous month, ED stay longer than 6 h before admission, receipt of more than 1000 mL of crystalloids during the 3 h preceding admission, terminal illness with a life expectancy of less than 3 months, and immediate need for advanced organ support. The study was approved by the local ethics committee (protocol number: 406, 10 March 2023) and conducted in accordance with the Declaration of Helsinki. Written informed consent was obtained from all participants or their legal representatives.

### 2.3. Study Protocol and Data Collection

All patients presenting to the ED with suspected infection underwent the laboratory testing required for calculation of the SOFA score to facilitate early identification of sepsis. At enrolment in the IMCU, demographic and clinical data were collected systematically, including age, sex, weight, and height, from which body mass index (BMI) was derived. Comorbidities and past medical history were ascertained through review of available medical records and used to calculate the Charlson Comorbidity Index (CCI). Vital signs required for the National Early Warning Score (NEWS) were recorded.

At admission to the unit, arterial blood gas analysis and venous blood sampling were performed, including complete blood count with differential, serum electrolytes, renal and liver function tests (including alanine aminotransferase, aspartate aminotransferase, total bilirubin, and serum albumin), coagulation parameters (prothrombin time-international normalised ratio, activated partial thromboplastin time, fibrinogen, and D-dimer), blood glucose, and inflammatory markers, including C-reactive protein and procalcitonin. APACHE II and SOFA scores were calculated from admission clinical and laboratory data using only variables obtained before initiation of any therapeutic intervention. An arterial catheter was inserted for invasive blood pressure monitoring and serial arterial blood gas analysis, including lactate measurement, and a urinary catheter was placed for accurate monitoring of urine output.

Thereafter, blood samples were collected daily at 08:00 for measurement of serum albumin concentrations. At each sampling time, fluid input and output over the preceding 24 h were recorded, allowing calculation of cumulative fluid balance (CFB) from admission according to the following formula:

$$Cumulative\;fluid\;balance=\frac{\left(cumulative\;fluid\;input\;\left[liters\right]-cumulative\;fluid\;output\;\left[liters\right]\right)}{Bodyweight\;[kg]}\;\times \;100$$

CFB, expressed as a percentage of body weight, was used as a surrogate measure of the volumetric effects of fluid therapy on intravascular volume status and was analysed in relation to serum albumin concentrations. Fluid input was recorded as the total volume administered (mL), whereas output consisted of urine volume measured by a urometer. Patient management, particularly fluid administration and norepinephrine use, followed a standardised internal protocol based on Surviving Sepsis Campaign guidelines [17]. Before study initiation, departmental meetings were held to standardise clinical practice.

No colloid solution, including human albumin, was used during the study period. Blood transfusions, when indicated, were administered according to clinical judgement and appropriately documented.

### 2.4. Serum Albumin Measurement

Serum albumin concentrations were measured on a Dimension Vista 1500 analyser (Siemens Healthcare Diagnostics Inc., Newark, DE, USA) using the bromocresol green colorimetric method. Results were expressed in g/dL at each prespecified time point.

### 2.5. Outcome

All-cause 30-day mortality was assessed during hospitalisation, at discharge, and, for patients discharged before the scheduled follow-up, by telephone interview with the patient or caregiver. When available, the timing and cause of death were also recorded.

### 2.6. Statistical Analysis

Continuous variables were summarised as mean (SD) or median (IQR), and categorical variables as counts and percentages. Between-group differences were assessed using Student’s *t* test or the Mann–Whitney U test, χ^2^ test, or Fisher’s exact test, as appropriate. Correlations were evaluated using Pearson’s or Spearman’s coefficients according to data distribution. The CFB, vasopressor use, and serum albumin trajectories were assessed using longitudinal mixed-effects models with a random intercept for each patient. Serum albumin was modelled as a continuous outcome with time point as a categorical variable. CFB denotes the absolute cumulative fluid balance at each time point, whereas ΔCFB denotes its change between consecutive reassessments and served as the primary time-varying exposure in all longitudinal models; absolute CFB was retained as a secondary specification. All multivariable models were adjusted for age, baseline severity (admission SOFA and APACHE II scores), comorbidity burden (CCI), and clinical acuity at reassessment (NEWS).

To assess whether vasopressor use modified the fluid–albumin association over time, we fitted a linear mixed-effects model with a random intercept for patient across reassessments, including a three-way interaction term between fluid exposure (ΔCFB), vasopressor use, and time point, with adjustment for baseline covariates. Marginal effects of fluid exposure on albumin were estimated for each combination of reassessment and vasopressor use, and the significance of the three-way interaction was assessed with a Wald test. A secondary landmark subanalysis at T1 evaluated whether early vasopressor use was associated with albumin concentrations at T3. For the purposes of the landmark analysis and all analyses stratified by vasopressor use, ‘early norepinephrine’ was operationally defined as initiation of norepinephrine at or before the first reassessment (T1), corresponding to within approximately 24 h of IMCU admission.

To evaluate whether vasopressor initiation changed the longitudinal trajectory of serum albumin, we fitted a linear mixed-effects model with a random intercept for the patient. Time (T0–T5 reassessments) was modelled as a continuous variable to estimate the slope of albumin change over the observation period. For patients who started vasoactive therapy, a time-dependent post-initiation indicator was defined (post = 1 from the first reassessment with vasopressor therapy onwards; 0 for previous observations and in patients who never received vasopressors). The model included time, the post-initiation indicator, and the time × post-initiation interaction term to estimate any change in albumin slope after initiation of vasoactive therapy. The coefficient for time represented the pre-initiation slope; the interaction term quantified the change in slope after vasopressor initiation. Standard errors were calculated using cluster-robust methods at the patient level.

The association between serum albumin concentrations and 30-day mortality was assessed in prespecified landmark analyses at T1, T3, and T5 using adjusted logistic regression, with albumin scaled per 0.5 g/dL decrease, and results are reported as odds ratios (ORs) with 95% CIs. To investigate whether albumin decline partially mediated the association between fluid exposure and 30-day mortality, a two-stage longitudinal mediation analysis with a lagged structure to preserve temporality was performed. Exposure was defined as the percentage increase in fluid balance during the preceding interval (ΔCFB_t−1_), and the mediator as albumin at the current time point (ALB_t_). In stage A (ΔCFB_t−1_ → ALB_t_), the association between fluid exposure and subsequent albumin was estimated using pooled linear regression with cluster-robust standard errors at the patient level, adjusted for reassessment, clinical severity, comorbidity burden, and time-varying vasopressor use. In stage B, the direct effect c′ on the outcome (ALB_t_, ΔCFB_t−1_ → 30-day mortality) was estimated using multivariable logistic regression with the same covariate set, whereas the total effect c (ΔCFB_t−1_ → 30-day mortality) was estimated using an analogous model without the albumin term. The indirect effect was then derived from the difference between the total effect (c) and the direct effect (c′) on the log-odds scale and exponentiated to obtain an OR. In all models, standard errors were clustered at the patient level. Comparison of the total and direct effects allowed estimation of the proportion of the association between fluid exposure and mortality that was potentially mediated by changes in albumin concentrations.

All tests were two-sided, with significance set at *p* < 0.05. Analyses were performed using Stata version 18.0 (StataCorp, College Station, TX, USA).

## 3. Results

A total of 389 patients with community-acquired sepsis admitted from the ED were enrolled in the study. Baseline characteristics at enrolment are reported in Table 1.

The mean age of the cohort was 71.6 years (SD 12.6); 62.2% of patients were male, and the overall comorbidity burden was substantial, with a mean Charlson Comorbidity Index of 5.4 (SD 2.9). Baseline illness severity was moderate, with a mean SOFA score of 4.5 (SD 2.1) and a mean APACHE II score of 12.8 (SD 4.8). Overall, 30-day mortality was 18.0% (70/389). A total of 1897 longitudinal measurements were available, with reassessment completion rates of 100% (389/389) at T1, 99.5% (387/389) at T2, 97.4% (379/389) at T3, 95.9% (373/389) at T4, and 94.9% (369/389) at T5.

Mean serum albumin concentrations declined progressively during the early reassessments, reaching a nadir at the third reassessment, whereas cumulative fluid balance increased during the initial phase of observation (Table 2). At every reassessment after enrolment, serum albumin was inversely associated with CFB, with consistently significant correlations throughout follow-up (Table 2). The proportion of patients receiving norepinephrine was highest at the early reassessments and decreased progressively thereafter (Table 2).

In longitudinal models, the association between fluid exposure and lower serum albumin concentrations remained significant after simultaneous adjustment for multiple indices of clinical severity. In the model including baseline SOFA, APACHE II, and NEWS, each one-percentage-point increase in ΔCFB was associated with a mean reduction of −0.029 g/dL in serum albumin (95% CI −0.038 to −0.020; *p* < 0.001). Similar results were observed when cumulative fluid balance was modelled as a time-varying covariate (β −0.030, 95% CI −0.037 to −0.024; *p* < 0.001). Vasopressor use was not independently associated with serum albumin concentrations in multivariable models. All severity indices (SOFA, APACHE II, and NEWS) were independently associated with lower albumin concentrations (Supplementary Table S1).

The three-way interaction between fluid exposure (ΔCFB), time, and vasopressor use on serum albumin trajectories was overall significant (χ^2^ = 21.65; *p* < 0.001), indicating that the relationship between fluid exposure and albumin was temporally modified in patients receiving vasoactive support (Supplementary Table S2). In particular, among patients receiving vasopressors, increasing ΔCFB was associated with a greater reduction in serum albumin during the early phase (T1–T3; β ranging from −0.037 to −0.057 g/dL per one-percentage-point increase in ΔCFB; *p* = 0.018), whereas at later reassessments this association was attenuated and tended to reverse direction (T4: β +0.065, *p* = 0.016; T5: β +0.072, *p* = 0.269). By contrast, in patients not receiving vasopressors, the effect of ΔCFB remained overall negative during the later phase (T3–T5, *p* ≤ 0.009), with no association observed in the first 24 h (T1, *p* = 0.891) (Supplementary Table S2).

In the landmark analysis centred on the first reassessment (T1), early vasopressor use (defined as treatment at T1) was not independently associated with serum albumin concentrations at T3 (approximately 72 h), after adjustment for albumin at T1, cumulative fluid exposure between T1 and T3, and indices of clinical severity (β −0.008 g/dL, 95% CI −0.091 to 0.076; *p* = 0.860). By contrast, cumulative fluid exposure between T1 and T3 remained independently associated with lower albumin concentrations at T3 (β −0.023 g/dL per one-percentage-point increase; *p* = 0.034), suggesting that cumulative fluid exposure is a key determinant of albumin decline.

**Figure 1:**
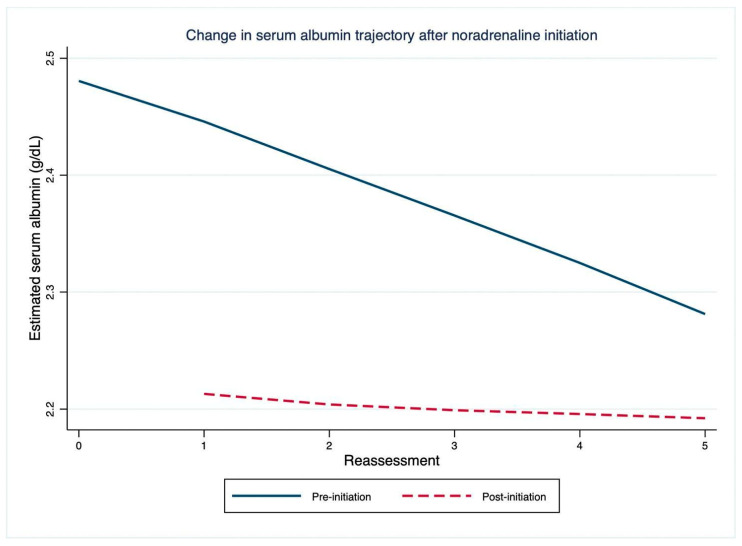
Longitudinal trajectory of serum albumin concentrations before and after initiation of vasoactive therapy, estimated using a linear mixed-effects model adjusted for baseline severity, comorbidity burden, and clinical acuity at reassessment. Before initiation of vasoactive therapy, serum albumin showed a progressive decline over time; initiation of treatment was associated with significant attenuation of the negative slope, with subsequent stabilisation of the albumin trajectory.

In the longitudinal model partitioned according to the period before and after norepinephrine initiation, serum albumin showed a progressive decline over time before the start of vasoactive therapy (Table 3). The pre-initiation slope was −0.043 g/dL per reassessment interval (95% CI −0.057 to −0.030; *p* < 0.001), indicating a steady decrease in albumin across observations. Norepinephrine initiation was associated with a significant attenuation of this trajectory. The interaction term between time and post-initiation status was positive and statistically significant (β +0.035 g/dL, 95% CI 0.008 to 0.063; *p* = 0.012), indicating a change in slope after initiation of vasoactive therapy. Combining the estimated coefficients, the post-initiation slope was approximately −0.008 g/dL per interval, consistent with a substantially flattened trajectory compared with the pre-initiation phase. This association remained significant after adjustment for admission SOFA and APACHE II scores, whereas the Charlson Comorbidity Index and NEWS were not significantly associated with longitudinal albumin change. Overall, initiation of vasoactive therapy was associated with a marked attenuation of the negative albumin slope over time (Figure 1).

Lower serum albumin concentrations at T3 were independently associated with higher 30-day mortality (Supplementary Table S3). Each 0.5 g/dL decrease in albumin was associated with a 49% increase in the odds of death (adjusted OR 1.49, 95% CI 1.05–2.12; *p* = 0.025), independently of cumulative fluid balance, baseline illness severity, comorbidity burden, clinical acuity, and norepinephrine use. A similar association was also observed at T1 (OR 1.37, 95% CI 1.02–1.89; *p* = 0.049), whereas at T5 the association was attenuated and no longer significant (OR 1.16, 95% CI 0.86–1.56; *p* = 0.338). Norepinephrine use at the corresponding time point was not associated with 30-day mortality (Supplementary Table S3).

Finally, in the lagged mediation analysis, a greater ΔCFB during the preceding interval (ΔCFB_lag(t−1)) was associated with lower albumin concentrations at the subsequent time point (β = −0.0417 g/dL per one-percentage-point increase in ΔCFB; *p* < 0.001), independently of time, clinical severity, comorbidity burden, and norepinephrine use. In the outcome model, lower albumin concentrations were associated with higher 30-day mortality (OR 1.26 per 0.5 g/dL decrease; *p* = 0.040), whereas ΔCFB_lag(t−1) remained positively associated with mortality both in the total-effect model (OR 1.095 per one-percentage-point increase; *p* = 0.013) and in the albumin-adjusted model (OR 1.076; *p* = 0.037). The point estimate of the indirect effect suggested that approximately 18.6% of the association between increasing fluid balance and mortality might be explained by lower albumin concentrations (indirect effect: OR 1.02 per one-percentage-point increase in ΔCFB), supporting the hypothesis that albumin decline represents a biologically plausible component of the pathway linking fluid exposure to outcome.

## 4. Discussion

In this prospective real-world cohort of patients with community-acquired sepsis, fluid exposure was independently associated with a progressive decline in serum albumin during the early phase of illness, suggesting that fluid therapy may contribute to the development of hypoalbuminaemia. Lower albumin concentrations were, in turn, associated with a higher risk of 30-day mortality, outlining a potential pathophysiological trajectory linking fluid exposure, loss of intravascular albumin, and adverse outcomes. In this context, early vasopressor initiation was associated with attenuation of albumin decline over time, indicating that the negative albumin slope was attenuated following vasopressor initiation. These findings are consistent with recent hypotheses in the literature, suggesting that albumin loss driven by inflammatory capillary leak and the dilutional effects of fluid resuscitation may be partially contained by early vasopressor use. Overall, these data support haemodynamic strategies aimed at limiting fluid exposure and introducing vasopressor support early in order to preserve intravascular albumin and potentially improve outcomes in patients with sepsis [13,14,15,16,18,19].

This study also provides new insights into the clinical interpretation of the pathophysiological mechanisms underlying sepsis.

First, the inverse association between CFB and serum albumin concentrations observed in our study provides empirical support for a mechanism that is often considered implicit in the pathophysiology of sepsis, yet remains surprisingly underdocumented by clinical data directly linking fluid administration to the development of hypoalbuminaemia [8,9,20]. However, it should be noted that multi-centre randomised trials such as ALBIOS have not demonstrated a mortality benefit from albumin supplementation in unselected sepsis populations [21], underscoring that the prognostic relevance of albumin decline does not necessarily translate into therapeutic benefit from exogenous albumin replacement and that the present findings must be interpreted within this broader evidential context. A key question is whether this relationship primarily reflects intravascular dilutional effects or pathological albumin loss due to increased transcapillary escape [8,9]. Several findings suggest that both mechanisms contribute; however, the magnitude of albumin decline observed in our cohort exceeded that expected from dilution alone, and the progressive strengthening of the correlation over time suggests that cumulative fluid exposure may amplify this process through progressive endothelial injury rather than through simple immediate haemodilution [18,22,23,24]. Experimental studies support this interpretation, showing that excessive crystalloid resuscitation may promote glycocalyx degradation and increase vascular permeability [23]. Even under physiological conditions, excessive fluid loading can exceed lymphatic drainage capacity, resulting in substantial loss of albumin from the intravascular compartment [24].

Second, the most clinically relevant finding was that vasopressor initiation was associated with immediate and sustained stabilisation of the albumin trajectory. Before vasopressor initiation, serum albumin showed a progressive decline, plausibly reflecting the combined effects of ongoing fluid loading and persistent capillary leak. At the time of vasopressor initiation, the slope was markedly attenuated, suggesting a durable stabilising effect on the intravascular compartment. Restoration of systemic vascular resistance may reduce transcapillary hydrostatic gradients, thereby limiting albumin extravasation across an already compromised endothelium. Moreover, achievement of haemodynamic targets with vasopressors rather than with additional fluid loading may favour a more restrictive fluid management strategy and an earlier transition towards negative fluid balance [25,26,27,28]. Norepinephrine may also improve microcirculatory perfusion and lymphatic drainage by restoring adequate perfusion pressure, thereby counteracting the interstitial albumin accumulation typically associated with aggressive fluid resuscitation [29]. These findings are consistent with recent randomised evidence supporting early vasopressor use and restrictive fluid strategies in sepsis (CENSER, CLASSIC, CLOVERS) [12,28,30], while the present study extends this evidence by examining albumin dynamics as a potential mechanistic intermediate linking fluid exposure to outcomes. Although direct evidence remains limited, it is plausible that early restoration of perfusion pressure contributes to stabilisation of the endothelial glycocalyx by reducing shear stress and ischaemia–reperfusion injury. In addition, a possible direct effect on the capillary–endothelial interface could help explain the ability of vasoactive agents to limit protein extravasation. Taken together, these findings suggest that early norepinephrine is not merely a temporary means of blood pressure support, but may represent an intervention capable of preserving intravascular albumin and limiting the harm associated with fluid overload, thereby providing a pathophysiological rationale for combined vasopressor–albumin haemodynamic strategies in sepsis.

Third, the three-way interaction between time, fluid exposure, and norepinephrine use indicated that the association between norepinephrine use and serum albumin trajectories evolved over the course of resuscitation. During the early phases of shock, patients treated with norepinephrine showed a stronger association between fluid exposure and albumin decline, consistent with more severe endothelial dysfunction, accelerated transcapillary leak, and greater fluid requirements. At later stages, this relationship tended to attenuate or even reverse, plausibly reflecting haemodynamic stabilisation, endothelial recovery, and changes in the clinical context of fluid administration. The positive coefficient observed at T4 in patients receiving vasopressors (β +0.065, *p* = 0.016) should be interpreted with caution and may reflect survivor bias, as only hemodynamically stabilised patients remained on vasopressors at this time point, as well as possible model instability due to reduced subgroup size at later reassessments rather than a genuine reversal of the fluid–albumin relationship. The landmark analysis further showed that early norepinephrine use did not independently influence subsequent albumin concentrations, suggesting that its effect operates mainly through modulation of haemodynamic status and fluid requirements rather than through a direct effect on albumin metabolism. Taken together, these findings support the interpretation that norepinephrine may promote preservation of intravascular albumin by reducing fluid exposure and restoring vascular tone. However, it should be noted that patients who received norepinephrine were, by definition, more hemodynamically unstable at the time of initiation, and adjustment for baseline SOFA and APACHE II scores does not fully capture the dynamic trajectory of haemodynamic deterioration. Therefore, residual confounding by indication cannot be excluded and represents the primary inferential limitation of the vasopressor-related analyses in this study

Fourth, the lagged mediation analysis, which reduces the risk of reverse causation and strengthens causal inference by focusing on the exposure–mediator–outcome sequence, showed that approximately 18.6% of the association between fluid exposure and mortality was mediated by albumin decline, suggesting that hypoalbuminaemia represents one of the mechanisms through which fluid overload contributes to adverse outcomes. Although haemodilution is the most immediate mechanism, experimental evidence indicates that volume overload increases capillary filtration and, in the presence of altered endothelial permeability, promotes interstitial oedema and progression of organ dysfunction [31,32]. However, the fact that mediation was only partial indicates that the prognostic impact of fluid exposure also involves albumin-independent pathophysiological pathways, including dilutional effects on coagulation, inflammatory activation with glycocalyx shedding, and impairment of microcirculatory perfusion related to elevated venous pressures [33]. In this context, albumin appears to be a mechanistically informative marker of fluid-related injury, although it cannot fully capture all of its biological consequences. The mediation analysis rests on several assumptions that merit explicit acknowledgement. The lagged structure preserves the temporal ordering of exposure (ΔCFB~t−1~), mediator (ALB~t~), and outcome (30-day mortality), reducing the risk of reverse causation. However, the analysis assumes the absence of unmeasured confounders of the exposure–mediator and mediator–outcome relationships, an assumption that cannot be verified in an observational design. Accordingly, the mediation estimate of 18.6% should be regarded as exploratory and hypothesis-generating rather than as a quantification of a causal pathway.

It is important to note that albumin is used in this study in two distinct analytical roles: as an independent predictor of 30-day mortality in the landmark analyses and as a potential mediator of the fluid exposure–mortality association in the mediation analysis. These roles are not mutually exclusive, but they do not imply that albumin decline is itself a causal determinant of outcome; rather, albumin decline may equally reflect the severity of endothelial dysfunction and capillary leak, functioning as a marker of disease severity rather than as an independent pathophysiological driver. Overall, our findings suggest that hypoalbuminaemia in sepsis is not merely an epiphenomenon of critical illness, but a pathophysiological node linking fluid exposure, endothelial dysfunction, and clinical outcomes. The observation that early vasoactive therapy attenuates albumin decline supports the hypothesis that restoration of vascular tone, and potentially reinforcement of the capillary–endothelial barrier, may help preserve the intravascular albumin pool and limit protein leakage, thereby defining a possible window of vascular competence during the early phase of sepsis [24,34]. In this setting, albumin may represent not only a prognostic biomarker but also a dynamic indicator of endothelial barrier function and of the balance between intravascular refilling and interstitial loss. These observations suggest that haemodynamic strategies aimed at limiting crystalloid exposure and introducing vasopressor support early may help preserve circulating albumin and reduce fluid overload-related harm. A major strength of this study lies in its prospective longitudinal design, with integrated serial measurements of albumin, fluid balance, and vasopressor therapy, which allowed exploration of the temporal dynamics of these phenomena during the early phase of sepsis. However, our findings should be regarded as hypothesis-generating and do not allow definitive causal conclusions. Prospective studies and randomised trials will be needed to determine whether early combined strategies based on norepinephrine and selective albumin use can improve clinical outcomes in patients with sepsis. Such trials should incorporate serial measurement of endothelial integrity biomarkers, including syndecan-1 as a marker of glycocalyx shedding, to elucidate the mechanistic pathways through which vasopressor-guided haemodynamic stabilisation may limit transcapillary albumin loss and whether this translates into measurable endothelial protection.

### Limitations

This study has several limitations. The single-centre design may have introduced bias related to local clinical practice. However, the management of patients with sepsis was standardised through an internal protocol based on guideline recommendations and periodic review by the IMCU medical team, which likely reduced these sources of bias. In addition, consistent with the existing literature, human albumin was not used in patient management, thereby limiting potential interference with serum albumin concentrations.

Other treatments administered according to guideline recommendations, such as corticosteroids and antibiotics, were not included in the study analyses.

Some patients discontinued follow-up during the observation period solely because of death. As in other longitudinal observational studies conducted in routine clinical practice, these patients were retained in the analyses.

Although used in clinical practice, data from more advanced ultrasound assessments or formal evaluation of fluid responsiveness were not collected. Dynamic indices of fluid responsiveness and echocardiographic measures of cardiac output might have allowed better characterisation of the hemodynamic context in which albumin decline occurred, potentially strengthening the interpretation of the fluid–albumin association.

The study observation period was limited to the first 5 days of treatment. Analyses of albumin trajectories over longer periods might provide a more complete understanding of intravascular and extravascular albumin behaviour.

Factors such as pre-existing nutritional status, enteral or parenteral nutrition, gastrointestinal losses, and fluid losses related to insensible perspiration were not considered. These variables may independently influence serum albumin concentrations and could represent unmeasured confounders of the observed fluid–albumin association, particularly in patients with prolonged hospitalisation or significant gastrointestinal dysfunction.

Detailed hemodynamic variables, including mean arterial pressure trajectories, serial lactate kinetics, and norepinephrine dose, were not included in the analyses. These parameters might have provided a more granular characterisation of the hemodynamic context in which albumin trajectories evolved and their absence represents a further source of residual confounding.

Systematic screening logs were not prospectively maintained, precluding construction of a participant flow diagram. The exclusion of patients with ED stays exceeding 6 h or pre-admission crystalloid volumes above 1000 mL may have selected a physiologically more homogeneous population, further limiting generalisability to patients with more substantial pre-hospital fluid loads.

Furthermore, the observational nature of norepinephrine administration introduces confounding by indication as a central inferential limitation of the vasopressor-related analyses. Patients who received norepinephrine were more severely ill at the time of initiation, and the observed attenuation of albumin decline following vasopressor use cannot be attributed to the intervention itself without accounting for the possibility that hemodynamic stabilisation is the operative mechanism. Randomised evidence is required to isolate the specific contribution of norepinephrine to albumin trajectory modulation.”

Finally, the influence of unmeasured confounders cannot be excluded. E-values calculated for the primary mortality association (OR 1.49 per 0.5 g/dL decrease in 72 h albumin) indicate that an unmeasured confounder would need to be associated with both the exposure and the outcome by a risk ratio of at least 2.34 for the point estimate and 1.28 for the lower bound of the confidence interval to fully explain the observed effect. These findings suggest that relatively modest unmeasured confounding could attenuate the observed association, consistent with the observational nature of the study.

## 5. Conclusions

In this prospective cohort of patients with moderate-severity sepsis treated in an IMCU, the present findings are hypothesis-generating and should not be extrapolated beyond comparable clinical settings. Cumulative fluid exposure was associated with a progressive decline in serum albumin, and this relationship was modulated over time by norepinephrine therapy. Initiation of vasopressor treatment was associated with marked attenuation of the downward albumin trajectory, suggesting that restoration of vascular tone may help limit capillary leak and support more restrictive fluid strategies. Albumin concentrations at 72 h also emerged as independent predictors of 30-day mortality and as a potential pathophysiological link between fluid exposure and clinical outcomes. Taken together, these findings suggest that albumin dynamics may represent a useful indicator of vascular barrier function during the early phase of sepsis. Prospective studies are needed to determine whether early haemodynamic strategies integrating norepinephrine use and selective albumin supplementation can translate into clinical benefit in patients with sepsis.

## Figures and Tables

**Table 1:** Demographic, medical history, and clinical characteristics of the study population.

Variables	
Patients, n (%)	389
Age, years, mean (SD)	71.6 (12.6)
Sex, n (%)	
Female	147 (37.8)
Male	242 (62.2)
Weight, kg, mean (SD)	75.1 (16.6)
BMI, mean (SD)	26.2 (5.5)
Medical history, n (%)	
Hypertension	247 (63.5)
Ischemic heart disease	52 (13.4)
Chronic heart failure	58 (14.9)
Stroke/TIA	33 (8.5)
COPD	49 (12.6)
Chronic kidney failure	73 (18.8)
Diabetes	105 (27)
Charlson Comorbidity Index, point, mean (SD)	5.4 (2.9)
Lactate, median (IQR)	1.7 (1.2–2.8)
SOFA score, point, mean (SD)	4.5 (2.1)
APACHE II, point, mean (SD)	12.8 (4.8)

**Table 2:** Mean serum albumin and cumulative fluid balance values across reassessments, with correlations between cumulative fluid balance and serum albumin. Percentage of patients receiving norepinephrine at each reassessment.

Reassessment	Patients	Albumin (SD)	CFB (SD)	Correlation Between ALB and CFB	*p*-Value	Norepinephrine, %
Enrolment	389	2.58 (0.53)	0	-	-	0
T1	389	2.32 (0.47)	2.08 (2.06)	−0.235	<0.001	40.1
T2	387	2.30 (0.52)	2.12 (3.34)	−0.270	<0.001	35.9
T3	379	2.24 (0.48)	1.87 (3.92)	−0.331	<0.001	22.4
T4	373	2.30 (0.55)	1.45 (4.32)	−0.318	<0.001	9.4
T5	369	2.32 (0.58)	1.20 (4.89)	−0.348	<0.001	6.5

**Table 3:** Change in serum albumin slope after initiation of vasopressor therapy: pre-initiation time was associated with a progressive decline in serum albumin (β = −0.043; *p* < 0.001). The time × post-initiation interaction (β = +0.035; *p* = 0.012) indicates a significant attenuation of the slope after vasopressor initiation. The estimated post-initiation slope was close to zero (approximately −0.008 per interval). This effect remained after adjustment for baseline severity.

Variable	Beta Coefficient	95% CI	*p*-Value
Time (pre-initiation slope)	−0.043	−0.057; −0.030	<0.001
Post-initiation (level shift)	−0.237	−0.325; −0.149	<0.001
Time × post-initiation	0.035	0.008; 0.063	0.012
SOFA	−0.037	−0.056; −0.018	<0.001
APACHE II	−0.014	−0.024; −0.003	0.010
CCI	−0.006	−0.022; 0.009	0.444
NEWS	0.002	−0.012; 0.015	0.799
Intercept	2.848	2.724; 2.973	<0.001
Random effects Patient-level intercept variance: 0.112 (95% CI 0.094−0.134) Residual variance: 0.146 (95% CI 0.128−0.165)			

## Data Availability

Data are only available on request due to privacy/ethical restrictions.

## Supplementary Materials

The following supporting information can be downloaded at: https://www.mdpi.com/article/10.3390/jcm15093203/s1, Table S1: Longitudinal mixed-effects models assessing the association between fluid exposure, vasopressor use, and serum albumin concentrations over time; Table S2: Patient-level longitudinal mixed-effects model assessing whether the association between fluid exposure (ΔCFB) and serum albumin concentrations is modified over time by norepinephrine use at individual timepoints; Table S3: Association between serum albumin concentrations at different reassessments (T1, T3, and T5) and 30-day mortality. Adjusted odds ratios (ORs) are reported for each 0.5 g/dL decrease in serum albumin, with corresponding 95% CIs and *p*-values. The model also included concurrent norepinephrine use at the same time point.

## Abbreviations

The following abbreviations are used in this manuscript:

APACHE II	Acute Physiology and Chronic Health Evaluation II
BMI	Body mass index
CCI	Charlson Comorbidity Index
CFB	Cumulative fluid balance
CI	Confidence interval
ED	Emergency department
IMCU	Intermediate care unit
IQR	Interquartile range
NEWS	National Early Warning Score
OR	Odds ratio
SD	Standard deviation
SOFA	Sequential Organ Failure Assessment
