# Supplementary material for: Early Norepinephrine Attenuates Fluid-Associated Albumin Decline in Sepsis: A Prospective Longitudinal Study
Source: J Clin Med. 2026 Apr 22;15(9):3203. doi: 10.3390/jcm15093203 (PMC13163598; doi:10.3390/jcm15093203)
Supplement: Supplementary file 1 [file jcm-15-03203-s001.zip › jcm-4252771-supplementary.pdf]

Supplementary Table S1. Longitudinal mixed-effects models assessing the association between fluid exposure, vasopressor use, and serum albumin concentrations over time.

	**Model A—ΔCFB**			**Model—CFB**		
Variables	Beta	95 CI%	*p*-Value	Beta	95 CI%	*p*-Value
Fluid exposure (%)	-0.029	-0.038 ; -0.020	<0.001	-0.030	-0.037 ; -0.024	<0.001
Norepinephrine	-0.021	-0.073 ; 0.031	0.431	-0.016	-0.067 ; 0.036	0.553
Baseline SOFA	-0.029	-0.049 ; -0.009	0.005	-0.032	-0.051 ; -0.013	0.001
APACHE II	-0.015	-0.025 ; -0.006	0.002	-0.013	-0.023 ; -0.004	0.005
NEWS	-0.011	-0.020 ; -0.003	0.011	-0.009	-0.017; -0.001	0.047
CCI	-0.013	-0.028 ; 0.002	0.087	-0.014	-0.028 ; 0.001	0.059
Age	0.003	-0.001 ; 0.006	0.138	0.003	-0.002 ; 0.005	0.335

Supplementary Table S2. Patient-level longitudinal mixed-effects model assessing whether the association between fluid exposure (ΔCFB) and serum albumin concentrations is modified over time by norepinephrine use at individual timepoints.

**Reassessment**	**Norepinephrine Use**	**Effect of ΔCFB on Albumin**	**95% CI**	***p*-Value**
T1	No	-0.002	-0.031 ; 0.027	0.891
T1	Yes	-0.037	-0.067 ; -0.006	0.018
T2	No	-0.020	-0.043 ; 0.003	0.088
T2	Yes	-0.057	-0.090 ; -0.023	0.001
T3	No	-0.029	-0.051 ; -0.007	0.009
T3	Yes	-0.056	-0.092 ; -0.020	0.002
T4	No	-0.035	-0.057 ; -0.014	0.001
T4	Yes	0.065	0.012 ; 0.118	0.016
T5	No	-0.036	-0.056 ; -0.016	<0.001
T5	Yes	0.072	-0.056 ; 0.200	0.269

Supplementary Table S3. Association between serum albumin concentrations at different reassessments (T1, T3, and T5) and 30-day mortality. Adjusted odds ratios (ORs) are reported for each 0.5 g/dL decrease in serum albumin, with corresponding 95% CIs and *p*-values. The model also included concurrent norepinephrine use at the same time point.

**Reassessment**	**Patients**	**Adjusted OR per 0.5 g/dL Decrease in Albumin**	**95% CI**	***p*-Value**	**Adjusted OR for Norepinephrine Use**	**95% CI**	***p*-Value**
T1	389	1.37	1.01–1.89	<0.001	0.97	0.53–1.73	0.909
T3	379	1.49	1.05–2.12	0.025	0.83	0.40–1.69	0.611
T5	369	1.16	0.86–1.56	0.338	1.51	0.49–4.63	0.470
